# Impact of acceptance and commitment therapy on work resilience among restaurant and hotel workers in tourism zones: a case of multivariate analysis

**DOI:** 10.1097/MD.0000000000050919

**Published:** 2026-10-02

**Authors:** Joy Nneka Uchenye Ejikeme, John Nwabueze Igabari, Daphney Mawila-Chauke, Moses Onyemaechi Ede, Glory Mmachukwu Nwakpadolu, Joy Oluchukwu Okoro, Blessing Ijeoma Attah, Eunice Ifenyinwa Ugwu, Joy Obiageli Oneli

**Affiliations:** a Department of Hospitality and Tourism Studies, University of Nigeria, Nsukka, Nigeria; b Department of Mathematics, Delta State University, Abraka, Delta State, Nigeria; c Department of Educational Psychology, University of Johannesburg, Soweto, South Africa; d Department of Agric/Vocational Education, College of Education, Michael Okpara University of Agriculture, Umudike, Nigeria; e Department of Home Economics and Hospitality Management Education, University of Nigeria, Nsukka, Nigeria; f Teleo Network International School of Theology, Pastoral Ministry Grace Training International Bible Institute, Duluth, Gambia.

**Keywords:** acceptance and commitment therapy, restaurant and hotel workers, tourism zones, work resilience

## Abstract

**Background:**

Cooperative efforts and the courage to withstand difficult times among workers in an organization enhance productivity. However, some employees become discouraged by hard times and unusual tasks in the organization, which leads to low resilience among workers. Empirical evidence shows that restaurant and hotel workers demonstrate low work resilience in the workplace. For this reason, this study investigated the impact of acceptance and commitment therapy (ACT) on work resilience among restaurant and hotel workers in tourism zones in Nigeria.

**Methods:**

Of the 189 restaurant and hotel workers who were recruited and assessed at the pretest stage, 122 participated in a 12-session ACT intervention and were assessed twice immediately after the intervention was implemented. Multivariate analysis was used to precisely examine how the intervention affected the dependent variable.

**Results:**

The findings showed that the ACT treatment group was more successful in improving workers’ resilience in the restaurant and hotel industries. The difference in mean stress scores between the pretest and posttest for male and female workers indicates a reduction in mean resilience scores for both groups. Male workers showed a smaller decrease in mean resilience scores compared with their female counterparts at the posttest stage.

**Conclusion:**

ACT intervention is an effective strategy for improving the resilience skills of restaurant and hotel workers with low resilience. Therefore, researchers and practitioners are encouraged to advance the practice and implementation of ACT tenets and principles in the workplace.

## 1. Introduction

The restaurant industry is one of the fastest-growing sectors,^[1]^ as the rise in precarious work schedules and multiple job-holding has coincided with a steady increase in the share of the family food budget spent on food prepared outside the home.^[2,3]^ Rising service demands in restaurants and the hotel industry have led to heavier workloads in recent years, resulting in over 11 million employees directly employed in food preparation and serving occupations in the United States.^[3]^ Similar statistics are lacking due to high turnover,^[4]^ which calls for research in Nigeria.

One important component of the tourism value chain is the rapidly developing hotel sector.^[5]^ Assessing the sector’s development potential also requires an understanding of workers’ psychological characteristics. The hotel business benefits from tourism growth.^[6,7]^ Tourism growth can occur both directly and indirectly as a region’s hotel stock expands.^[8,9]^

According to a report, the hospitality sector created more than 295 million jobs globally.^[10]^ In 2021, Nigeria’s industry provided more than 2.4 million job opportunities for the country’s large population.^[11]^ Chukwudi, Yetunde, and Aniekan^[12]^ have reported high employee turnover in the hospitality and tourism industry. This is a serious problem, as replacing skilled staff in this sector is very difficult given the high costs of recruitment and selection, as well as training and development.^[4]^ Employees work long hours in poor working conditions, including low salaries.^[4]^

Work hazards, pressures, and stress not only affect organizational well-being but also pose significant health risks for organizational staff. Stress at work can be problematic when workers frequently encounter contradictory demands from supervisors, customers, and the organization.^[13]^ Despite this, research on stress in the hospitality sector is lacking.^[14]^ This has led to low-quality customer service delivery,^[15]^ unproductive work on the job,^[16]^ a decrease in job performance quality,^[17]^ increased fatigue,^[18]^ and withdrawal.^[19]^ In this context, there is a dearth of knowledge about the current situation among employees in the hospitality sector, as well as a very small body of literature of this kind.^[14]^ Unfortunately, hotel employees who deal with a heavy workload feel less satisfied with their jobs and are considering quitting because of work stress.^[14]^ This underscores the need for researchers to seek ways to help restaurant and hotel workers cope with these unfortunate situations. Although this study investigated how psychological intervention could help employees in the hospitality industry manage work stress, it is noteworthy that past studies had established positive effects on other worker populations but not on hospitality workers in the tourist zone in Nigeria. To the best of our knowledge, none of the reviewed qualitative and quantitative studies have addressed this issue.

Resilience keeps employees focused, determined, and engaged despite the challenges. Resilience has become an emerging and critical issue in the hospitality industry, given the higher work demands in a contemporary, competitive society. It is a critical component of employee success and well-being, as well as organizational performance, particularly in high-pressure industries such as hotels, restaurants, and public kitchens. This is apparent. Indeed, resilience within the Nigerian hospitality industry is lacking.^[20,21]^ Meanwhile, employees are required to be consciously resilient to keep the hospitality industry relevant.^[20,22]^ Accordingly, the hospitality industry is recommended to focus on resilience training for employees. This would create opportunities for the exploration and exploitation of new ideas, such as entrepreneurial resilience and flexible working arrangements.^[23,24]^ Therefore, this study is anchored in providing resilient training for employees to improve customer-oriented services, not only because of reduced and depleted services but also because of a lack of strong resilience capacities in the Nigerian hospitality industry.^[20]^ It is one of the critical problems facing most hospitality businesses in southeastern Nigeria.^[20]^ If nothing is done to challenge and dispute the thoughts responsible for unconscious and low resilience in the hospitality workplace, the teeming 2.4 million workers and employers stand a chance of losing their jobs. Our firm position is that if there is impactful resilience and employees bounce back with high resilience through exposure to coping strategies,^[25]^ normalcy will be returned. Therefore, the main focus is on which coping mechanisms would have predictive positive results on employees’ efficacy levels.^[26]^

Past studies have recommended a cognitive-behavioral approach for individuals with low resilience to improve job efficiency and effectiveness in workplaces. One of the mindfulness-based workplace training interventions is acceptance and commitment therapy (ACT),^[27]^ a psychological intervention that combines commitment techniques, behavior modification, and awareness- and acceptance-based approaches to enhance psychological flexibility.^[28]^ Instead of directly trying to change clients, ACT aims to help them experience their experiences in various ways, increasing psychological and behavioral flexibility in situations where avoidance is prevalent and prevents clients from living their normal lives. ACT has become a resourceful alternative for employees to increase the quality of service delivery in workplace settings. Furthermore, it has been discovered that ACT interventions enhance worker performance and well-being, fostering greater creativity at work.^[29,30]^

The goal of ACT therapies, whether provided in clinical or nonclinical settings, is to help individuals develop psychological flexibility, a broad skill. Psychological flexibility is the ability to persist in or change behavior in the face of competing psychological influences, guided by values and goals that depend on the situation at hand.^[31]^ This skill can be utilized in various contexts: increased openness to unpleasant feelings and thoughts at work, the ability to bring present-moment awareness to tasks, internal experiences, and external interactions, and engagement in work-related behaviors that align with overall objectives and values.^[29,32]^ In recent years, ACT has emerged as a representative and effective psychotherapy due to its pragmatic philosophy of functional contextualism and theoretical underpinnings in relational frame theory.^[33]^ Through 6 fundamental processes, ACT primarily seeks to increase psychological flexibility so individuals can effectively adapt to challenges, guided by cognition rather than by futile attempts to control or eradicate undesirable thoughts, feelings, and experiences.^[34]^ Acceptance, self as context, values, being present, cognitive defusion, and committed action are the 6 processes.^[28,35,36]^ Various techniques, including mindfulness, cognitive defusion, metaphorical use, personal values clarity, and values-based behavioral activation, are used to cultivate psychological flexibility.^[37]^

When used in the workplace, ACT often comprises self-help books and group skills training workshops.^[38]^ Empirical evaluations of ACT interventions have been conducted across various organizational contexts, including educational, social care, and healthcare settings.^[3,39]^ A few studies have demonstrated the effectiveness of self-help or web-based ACT programs for diverse occupational samples.^[40,41]^ The global proliferation of ACT trials has generated empirical research in multiple regions, with most studies reporting positive outcomes in favor of ACT. However, research in Sub-Saharan Africa, including the Nigerian context, remains scarce.^[42]^ To date, no contraindications or iatrogenic effects have been reported, although these have not been extensively studied. Some studies have found ACT less effective than a control group in certain comparisons, while others have shown different change trajectories between ACT and the control condition. The impact of ACT on the hospitality industry in Nigeria remains unknown. Resilience literature in Nigeria, particularly among hospitality workers, is inadequate. Studies on resilience in other nations and populations, such as healthcare professionals, are documented but unevenly distributed, with industrial assessments underrepresented. This study aims to investigate the effect of ACT on the resilience of restaurant and hotel workers.

## 2. Methods

### 2.1. Ethical approval and participants

This study was registered with the UMIN Clinical Trial Registry with Unique ID Number: UMIN000061444. The study was approved by the Strategic Contacts, Ethics, and Publication Committee of the University of Nigeria with approval number UNN/EC/STA/010/FE=RC101.

A total of 122 workers participated in this study. The research team received permission from the lead researcher’s university to conduct the study. After obtaining permission, we visited 29 restaurants and 14 hotels to seek management approval for willing staff to participate. This was done to prevent any repercussions for the workers involved. Flyers were then distributed to 189 workers, with 122 responding positively and expressing interest in joining the study. The demographic information of the participants is presented in Table 1. Table 1 showed no significant differences in the demographic characteristics of participants in terms of working experience (χ^2^ = 4.650, *P* = .199), place of work (χ^2^ = 0.033, *P* = .856), and gender (χ^2^ = 0.033, *P* = .856) between the treatment group and the control group.

**Table 1:** Demographic information of the participants.

Demographics	Treatment group	Control group	χ^2^	*P*-value
Work experience				
1–5 yr	7 (33.3%)	14 (66.7%)		
6–10 yr	11 (42.3%)	15 (57.7%)	4.650	.199
11–15 yr	23 (59.0%)	16 (41.0%)		
16–20 yr	20 (55.6%)	16 (44.4%)		
Place of work				
Restaurant	33 (49.3%)	34 (50.7%)		
Hotel	28 (50.9%)	27 (49.1%)	0.033	.856
Gender				
Male	31 (50.8%)	30 (49.2%)	0.033	.856
Female	30 (49.2%)	31 (55.0%)		

The sample size’s statistical power was confirmed using G*Power. Detailed information regarding the sample calculation is illustrated in Figure 1.

**Figure 1.**
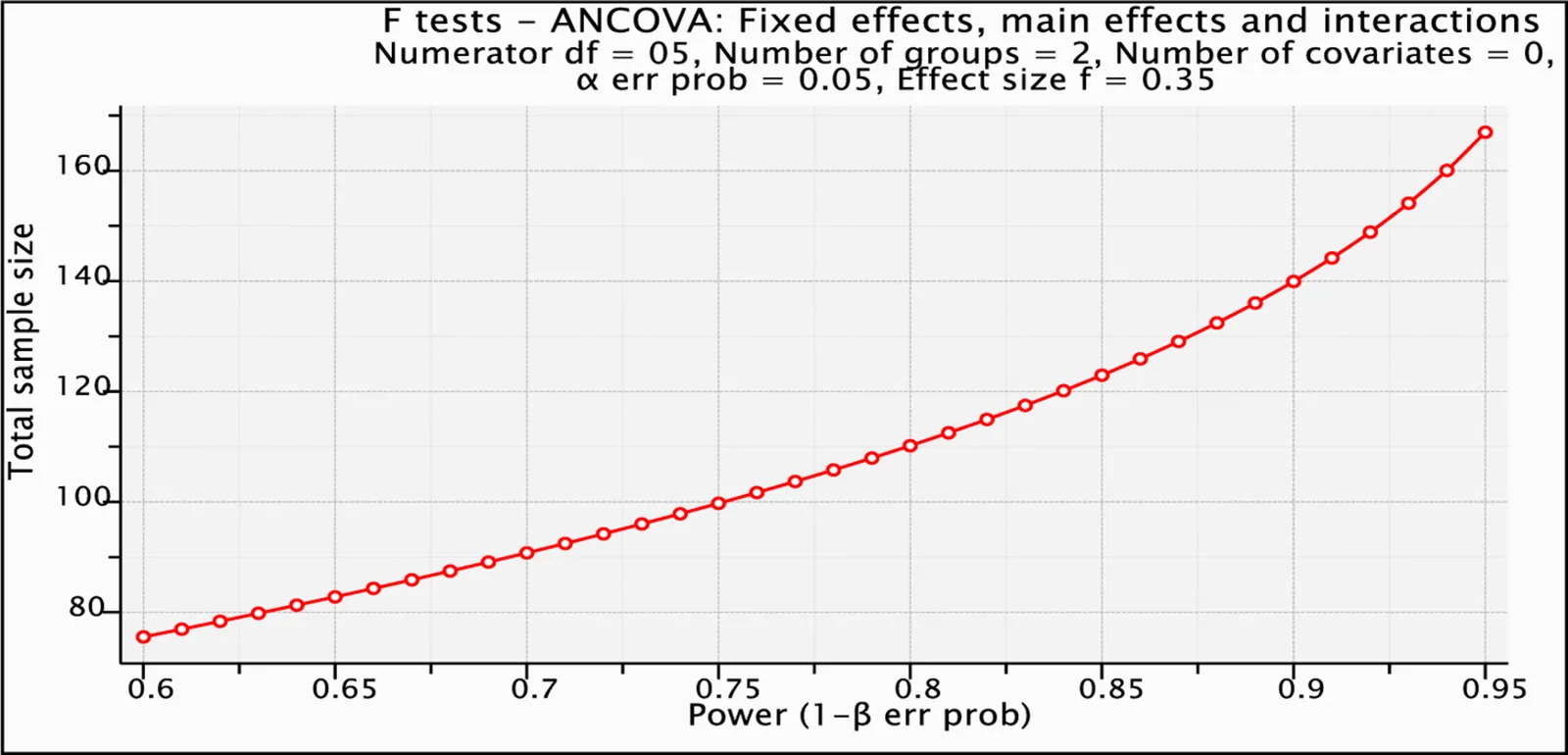
Sample size calculations. ANCOVA = analysis of covariance.

### 2.2. Dependent measure

The Ego Resilience Scale^[43]^ was used to assess participants’ career resilience. The 14 items on the measure use a 4-point Likert scale, with 1 denoting “does not apply at all,” 2 denoting “applies slightly, if at all,” 3 denoting “applies somewhat,” and 4 denoting “applies very strongly.” A score of 47 to 56 indicated very high resilience, a score of 35 to 46 indicated strong resilience, a score of 11 to 34 indicated poor resilience, and a score of 0 to 10 indicated very low resilience. The sum of the item scores determines the overall score, which represents the individual’s level of ego resilience. Higher scores indicate greater ego resilience. In the study by Block and Kremen,^[43]^ strong reliability was demonstrated, with an internal consistency of 0.76 among late adolescents and young adults. Because the scale addresses constructs that are crucial to the current research, including problem-solving skills, flexibility, and emotional regulation, it was selected for this investigation. In the current setting, a reliability test yielded an internal consistency of 0.72.

### 2.3. Procedure

The participants who showed interest were screened for eligibility based on the following inclusion criteria:

1. Scoring above a certain threshold on the dependent measure.
2. Being a staff member at a restaurant or hotel.
3. Willingness to participate.
4. Having worked at a restaurant or hotel for over a year.

Exclusion criteria included:

1. Not meeting the threshold score.
2. Participation in other psychological interventions.
3. Receiving clinical or nonclinical interventions in the past year.
4. Being subject to corporal punishment or having a letter of inquiry at work.

The excluded staff (n = 67) were excluded because some did not meet the inclusion criteria (n = 22), 28 staff voluntarily declined to participate, and 17 staff provided other reasons (see Fig. 2). The 122 eligible participants were briefed on slips of paper, which were wrapped and mixed in a plastic bowl. Each participant was instructed to pick one wrapped slip without replacement. The slips were divided into 2 forms: ACT and CG. ACT represented the intervention group, while CG represented the control group. Sixty-one participants who selected ACT were assigned to the intervention group, while 61 participants who chose CG were assigned to the control group. The participant flow was further described in Figure 2. The intervention group received a 12-session workplace training manual known as the ACT program. This program aimed to enhance the psychological flexibility of restaurant and hotel workers with low resilience. The first session was used to establish rapport among the participants, creating a positive psychological environment. The second session addressed building resilience in the workplace. The third session focused on the relationship between resilience and values. At this point, what a meaningful life is was discussed; participants were asked what was meaningful to them, and this information was used as a guide to modify participants’ behavior. In this third session, discussions focused on how committed action could lead to resilience. Training on psychological flexibility was given in session four. It also involved teaching how to manage difficult times that cause painful thoughts, feelings, and unproductive behaviors. Managing such difficult times could prevent a negative impact on life. At this point (fourth session), the important role of psychological presence and self-control in restaurants and hotels was discussed in depth. The fifth session was about how to become actively and psychologically involved in events in hotel programs, meeting the needs of customers. This involved directly, completely, and without unnecessary protection, engaging with psychological events. It also meant being a fully conscious human being in defused, open, and undefended contact with the present. The sixth session focused on how to observe ideas or thoughts instead of thinking about them, that is, observing thoughts rather than becoming sucked into them. They were trained to recognize thoughts for what they are rather than for what they appear to be. After this, a seventh session was introduced. This session addressed the importance of observing the self. The essence is to observe and accept all changing experiences. In the eighth session, the relationship between values, goals, and actions was the focus. Participants engaged in practice exercises on how to do more work on diffusion and acceptance. The ninth session focused on training participants to accept and be willing to get in touch with their values. Session 10 addressed how to identify barriers to values, goals, and action recognition. This was followed by developing mindfulness skills to remove barriers. The 11th session dealt with understanding and focusing on the directions of work and personal values. The 12th session was in the termination stage.

**Figure 2.**
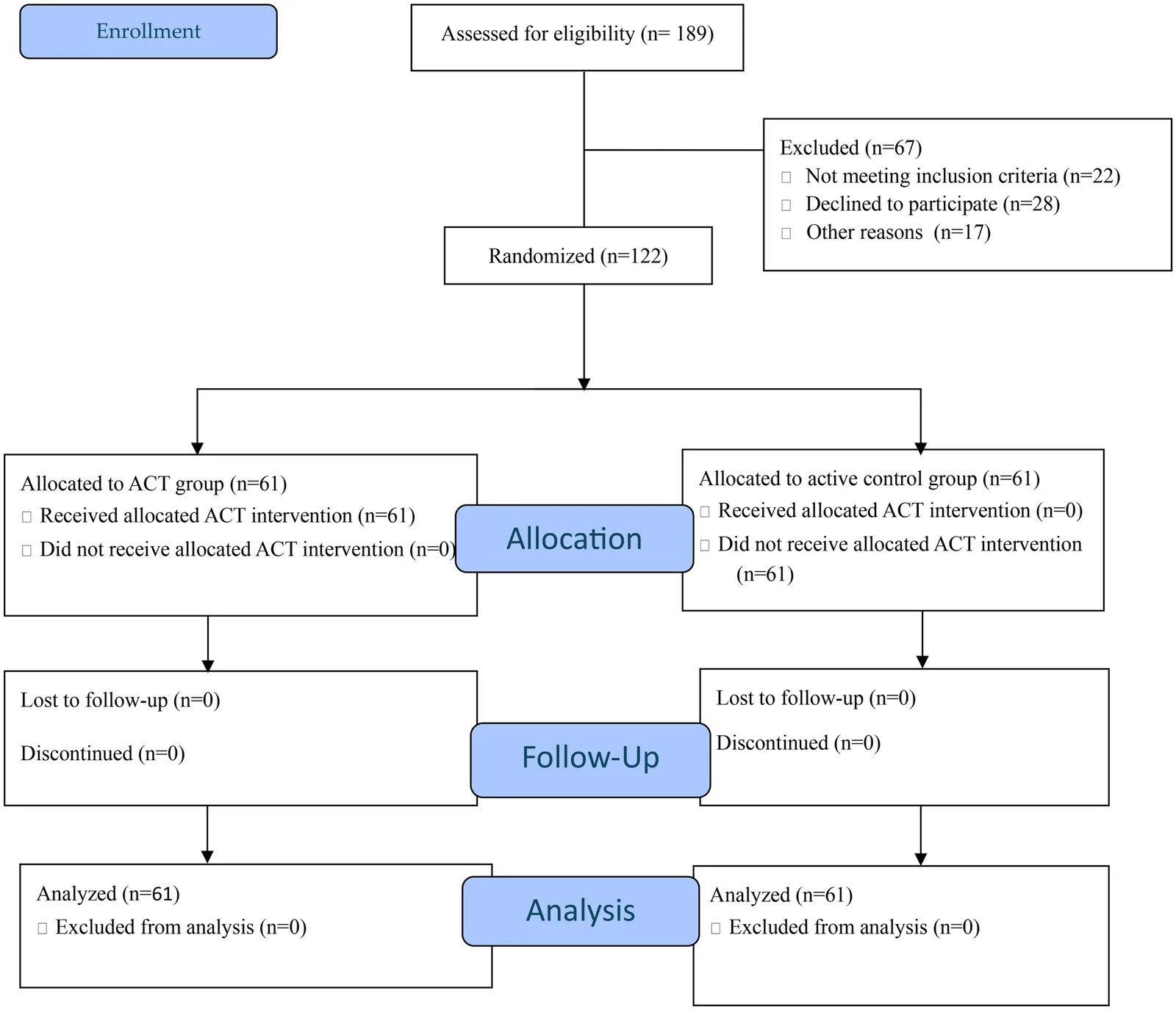
Participant flow diagram describing how participants are grouped and excluded. ACT = acceptance and commitment therapy.

Participants in the active control group received traditional counseling on work-life, which also lasted for 12 sessions, similar to those in the intervention group. A different career counselor implemented the sessions, and the active control group served as the control condition.

After delivering the program, the research team, with the help of treatment assessors, distributed copies of the dependent measure to the participants. This postintervention test aimed to determine whether the workers’ resilience had improved due to exposure to the ACT intervention. Gathering the postintervention data lasted for 6 days, as some participants left the treatment venue immediately after the program. Assistants were sent to their duty posts to collect their responses.

Two months later, participants were recalled for a follow-up test. Copies of the dependent measure were distributed for the third time to both groups. Upon receiving the pretest, posttest, and follow-up test data, the researchers blinded those who conducted each test, respectively.

### 2.4. Therapist and integrity check

The therapist who delivered the ACT intervention was a counseling psychologist with 9 years of experience practicing counseling psychology in occupational settings. The role was to follow the program steps and ensure the sessions were delivered correctly. Another expert was recruited and contracted to counsel members of the control group. The role was to assist the control group members. Both groups were assigned treatment assessors to ensure that the expected outcome, focusing on the set objective, was achieved.

### 2.5. Data analysis

The data collected at Time 1, Time 2, and Time 3 were statistically evaluated using the Statistical Package for the Social Sciences (SPSS), version 20 (IBM Corp., Armonk). Multivariate analysis was used to precisely look at how the intervention affected the dependent variable. To measure the effect of the intervention, the data were subjected to the partial eta squared statistical approach. Additionally, crosstabulation analysis was used to describe the demographics of the participants.

## 3. Results

After assessing 189 participants for eligibility, 122 staff members were recruited into the study and randomized into 2 groups (intervention group: 61 participants and control group: 61 participants); 67 staff were excluded for different reasons. Some were not able to meet the inclusion criteria (n = 22), 28 staff voluntarily declined to participate, and 17 staff provided other reasons. The 122 staff members who participated in the study were assessed 3 times.

The results in Table 2 show the pretest, posttest, and follow-up mean resilience scores of workers exposed to the ACT group and those not exposed to it. Results showed that the group exposed to the treatment had a pretest stress mean score (*x̄* = 33.58, standard deviation [SD] = 4.09), a posttest mean score (*x̄* = 41.86, SD = 2.73), and a follow-up mean score (*x̄* = 42.25, SD = 3.25). The results also show that the group not exposed to treatment had a pretest mean score (*x̄* = 33.25, SD = 3.50), a posttest mean score (*x̄* = 34.05, SD = 3.48), and a follow-up mean score (*x̄* = 34.05, SD = 3.48). The mean score differences between the pretest, posttest, and follow-up for the treatment group and the control group at the posttest and follow-up stages, respectively, compared with the pretest results, demonstrate that the ACT treatment group was more successful in improving the work resilience levels of workers in the restaurant and hotel industries.

**Table 2:** Descriptive statistics comparing mean and standard deviation at 3 times of measurement: before, after intervention, and follow-up.

Group	Pretest (M ± SD)	Posttest (M ± SD)	Follow-up (M ± SD)
Acceptance and commitment therapy	33.58 ± 4.09	41.86 ± 2.73	42.25 ± 3.25
Control	33.25 ± 3.50	34.05 ± 3.48	34.05 ± 3.48

SD = standard deviation.

The results in Table 3 show the influence of gender on the work resilience of workers. The results showed that the pretest, posttest, and follow-up mean resilience scores of male workers in the restaurant and hotel industries were *x̄* = 33.52, SD = 3.79; *x̄* = 41.25, SD = 3.04; and *x̄* = 42.35, SD = 3.69. The results also show that the pretest, posttest, and follow-up mean resilience scores of female workers in the restaurant and hotel industries were *x̄* = 33.63, SD = 4.44; *x̄* = 42.48, SD = 2.25; and *x̄* = 42.15, SD = 2.78. The difference in mean stress scores between the pretest and posttest for male and female workers indicates that the mean resilience scores of both male and female workers increased, with male workers having a lower mean resilience score difference than their female counterparts at the posttest stage.

**Table 3:** Comparing mean and standard deviation at 3 times of measurement: before, after intervention, and follow-up based on gender.

Group	Pretest (M ± SD)	Posttest (M ± SD)	Follow-up (M ± SD)
Acceptance and commitment therapy			
Male	33.52 ± 3.79	41.25 ± 3.04	42.35 ± 3.69
Female	33.63 ± 4.44	42.48 ± 2.25	42.15 ± 2.78
Control group			
Male	33.15 ± 3.69	34.94 ± 3.99	34.94 ± 3.99
Female	33.34 ± 3.36	33.18 ± 2.70	33.18 ± 2.70

SD = standard deviation.

Results in Table 4 show that there was no significant difference in the reduction of resilience of workers exposed to ACT and those in the control group at baseline, *F*(1, 121) = 0.228, *P* = .634, η^2^_*p*_ = 0.002. The posttest results showed a statistically significant effect of ACT on the work resilience of workers in the restaurant and hotel industries, *F*(1, 121) = 198.213, *P* ≤ .01, η^2^_*p*_ = 0.627. This indicates that participants’ responses to work resilience at different times of measurement are significantly different. The results also indicate that work resilience scores of workers in the restaurant and hotel industries were not significantly influenced by gender, *F*(1, 121) = 0.217, *P* = .642, η^2^_*p*_ = 0.002. It further indicated that there was a significant interaction effect of group and gender, *F*(1, 121) = 7.250, *P* = .642, η^2^_*p*_ = 0.058 (see Fig. 3). The follow-up results reveal that the effectiveness of exercise-based counseling intervention was maintained over time, *F*(1, 121) = 183.632, *P* ≤ .01.

**Table 4:** Multivariate analysis of the effect of ACT on resilience.

Source	Dependent variable	Type III sum of squares	df	Mean square	*F*	Sig.	Partial eta squared
Corrected model	ERSPretest	4.025	3	1.342	0.091	0.965	0.002
	ERSPosttest	1930.525	3	643.508	68.650	0.000	0.636
	ERSFollowUptest	2102.386	3	700.795	62.882	0.000	0.615
Intercept	ERSPretest	136,153.747	1	136,153.747	9250.578	0.000	0.987
	ERSPosttest	175,778.377	1	175,778.377	18,752.084	0.000	0.994
	ERSFollowUptest	177,572.083	1	177,572.083	15,933.414	0.000	0.993
Group	ERSPretest	3.351	1	3.351	0.228	0.634	0.002
	ERSPosttest	1858.009	1	1858.009	198.213	0.000	0.627
	ERSFollowUptest	2046.507	1	2046.507	183.632	0.000	0.609
Gender	ERSPretest	0.681	1	0.681	0.046	0.830	0.000
	ERSPosttest	2.037	1	2.037	0.217	0.642	0.002
	ERSFollowUptest	28.893	1	28.893	2.593	0.110	0.021
Group × Gender	ERSPretest	0.041	1	0.041	0.003	0.958	0.000
	ERSPosttest	67.962	1	67.962	7.250	0.008	0.058
	ERSFollowUptest	18.454	1	18.454	1.656	0.201	0.014
Error	ERSPretest	1736.772	118	14.718			
	ERSPosttest	1106.109	118	9.374			
	ERSFollowUptest	1315.067	118	11.145			
Total	ERSPretest	137,933.607	122				
	ERSPosttest	178,748.929	122				
	ERSFollowUptest	180,977.908	122				
Corrected total	ERSPretest	1740.796	121				
	ERSPosttest	3036.633	121				
	ERSFollowUptest	3417.453	121				

ACT = acceptance and commitment therapy, ERS = Ego Resilience Scale.

**Figure 3.**
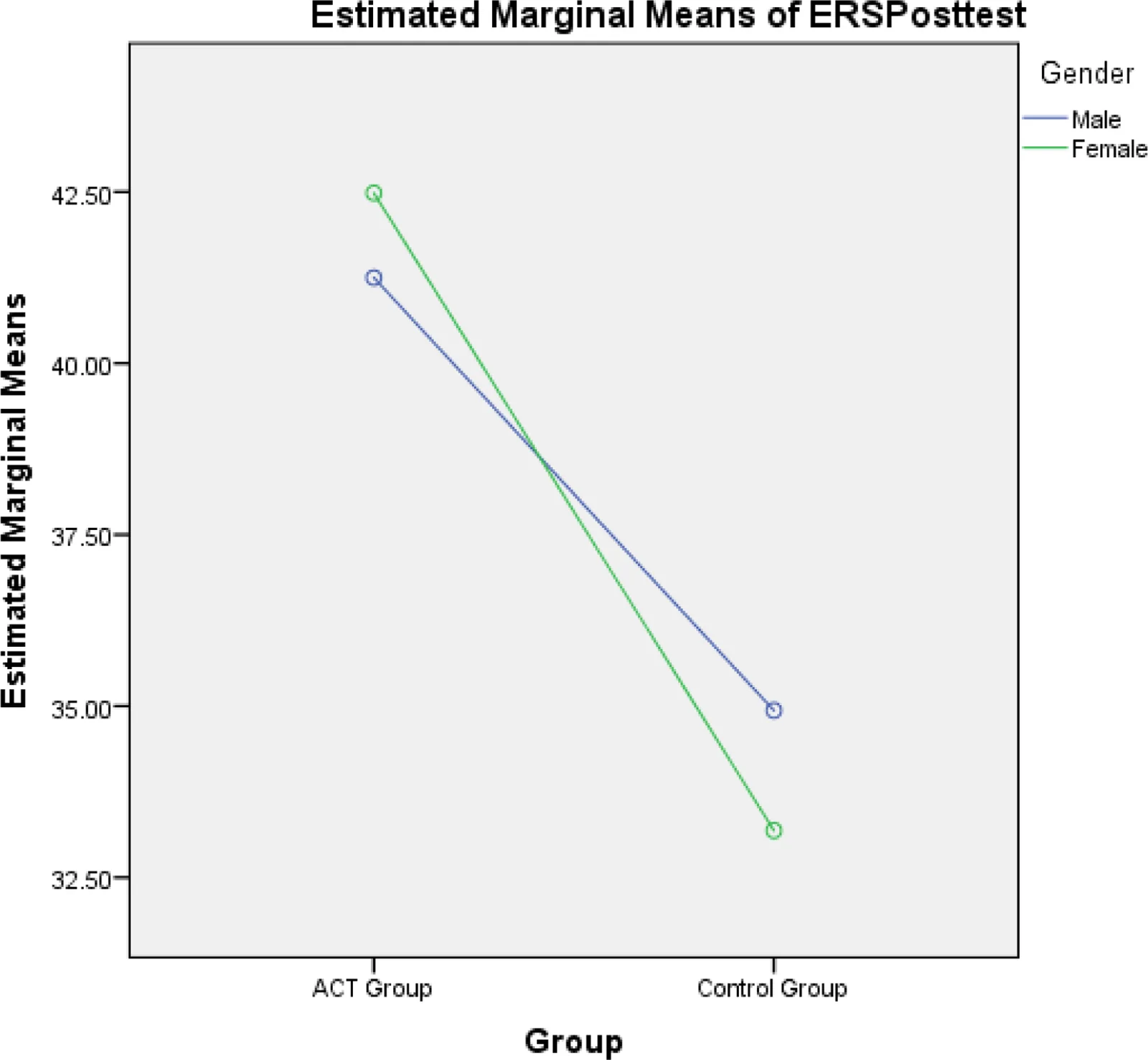
Interaction effect of group and gender. ACT = acceptance and commitment therapy, ERS = Ego Resilience Scale.

## 4. Discussion

The motivation for this study was to determine how ACT can effectively increase the work resilience of workers in the restaurant and hotel industry, given the unfavorable working conditions, poor economy, and insecurity in Nigeria. The outcome of this study was positive, showing that ACT significantly increased the work resilience of workers in the restaurant and hotel industry. A few weeks later, the effectiveness of ACT was evaluated again, and the results remained significantly positive, with sustained improvement.

Regarding ACT’s effectiveness in building resilience, it may be argued that the desire to avoid unpleasant internal experiences, such as thoughts and emotions, is what keeps them persistent. According to this approach, employees in hotels and restaurants follow their training because they have been taught that controlling and altering stress-inducing thoughts is necessary to overcome poor performance. This increases psychological flexibility and reduces work avoidance, which is the main objective of ACT.^[44]^ This kind of treatment not only increases workers’ acceptance of mental experiences, cognitive dissociation, flexibility, and appreciation of value, but also increases self-awareness among workers in the hospitality industry. Thus, negative ideas are treated with less gravity rather than being completely eradicated.^[45]^ The willingness to go through whatever level of discomfort is a vital component of acceptance, which is always a beneficial process.^[46]^

To understand the comparison between the present findings and past studies, we reviewed ACT literature. Many studies shared similar findings. For example, a related empirical study showed that ACT significantly increased patients’ resiliency, with this effectiveness still being significant at the follow-up level.^[47]^ Another related study found that acceptance and commitment treatment significantly improved the resilience of medical staff in Hamadan, with these effects persisting during the follow-up period.^[48]^ The best way to boost resilience is through acceptance and commitment training to improve skills.^[49]^ Given the current and past evidence, resilience is a key concept in understanding the adaptation process and learning adaptability techniques.^[50]^ Tugade and Fredrickson demonstrated that resilience gives rise to the idea that painful experiences may be changed, altered, or even eliminated.^[51]^

Further empirical review of past studies confirmed that the present findings are consistent with research conducted in Iran.^[52,53]^ This is also consistent with studies,^[54–56]^ which concluded that online training of therapy based on acceptance and commitment significantly increased the resilience of nurses suffering from COVID-19, which is consistent with the results of the current research. The results of Haddadi et al^[57]^ showed that ACT significantly improved nurses’ resilience.

The agreement indicates that resilience is a significant psychological structure necessary for perceiving excitement, motivation, and behavior.^[48]^ People with high self-resilience typically exhibit more positive emotions, enthusiasm, self-confidence, and psychological adjustment due to their increased flexibility. Those lacking resilience are conservative and rigid, making them more prone to act inconsistently under trying circumstances. Therefore, individuals who have received psychological training and have a practical attitude toward acceptance and commitment are more flexible and resilient when faced with difficulties. Resilience is increased through mindfulness practices, helping individuals alter unhelpful thought patterns, negative behaviors, and health-related behaviors.^[58]^ The studied literature spans multiple nations with diverse work situations and cultures, highlighting the beneficial effects of ACT on resilience and its intercultural penetration.

The findings also showed that gender had no discernible impact on employees’ work resilience scores in the restaurant and hotel sectors. ACT intervention is more directly shaped by job-specific demands (such as hotel and restaurant services) than by gender identity. Gender does not determine work resilience or control the impact of ACT intervention on workers’ resilience in hotels and restaurants. The main sources of psychological flexibility in service roles include problem-solving abilities, distress tolerance, and work experience. Along with optimism, hope, and self-efficacy, resilience is a component of psychological capital that is primarily influenced by individual characteristics, prior experiences with hardship, and cognitive evaluation, rather than by gender. This is understandable given that psychological traits such as resilience are essential for adapting to and recovering from work-related stress in a comparable manner across demographic groups under similar employment circumstances.^[45]^

The study’s conclusion is that shared occupational identity replaces gendered socialization. In contrast to classic Social Role Theory, which contends that gender socialization causes men and women to develop different coping mechanisms, the hospitality industry cultivates a robust subculture of cooperation and group perseverance.^[59]^ Given this difference, the management of hotels and restaurants should put team cohesion above gender-specific behavioral characteristics in order to unify how employees deal with challenges at work.

The results demonstrated why gender is not the driving factor. When it comes to determining employee resilience in the hospitality industry, other contextual and organizational elements are significantly more important than gender. Organizational support and leadership, which have a significant impact on resilience, are among the determinants. Here, resilience is directly enhanced by perceived supervisor support and psychological safety. Individual coping strategies or external stressors (such as off-shift family duties or structural pay/role inequities) can still differ, even though resilience capacity may be identical across genders.

## 5. Limitations

The study’s limitations include potential measurement bias introduced by the questionnaire used for data collection, which may limit its generalizability, and the short follow-up period, which may have restricted the investigation of long-term effects.^[48]^ A major methodological limitation is the absence of any baseline mental health assessment. Psychological conditions such as depression, anxiety, burnout, or other psychiatric disorders may substantially influence work resilience and may also improve following ACT, independent of occupational functioning. Without screening for these important potential confounders, it is difficult to determine whether the observed improvements are attributable specifically to the intervention. Given that no mental health assessment was performed before enrollment, we acknowledge it as a major limitation. Finally, it was expected that sample size would have considered the corresponding population.^[60]^

## 6. Strengths

We have mentioned the major limitations of this study. However, this study has made huge contributions to the literature. A past study noted how a small sample size affected the generalizability of the study and could increase the likelihood of type II errors,^[59]^ but the present study recruited 122 participants to overcome such threats. As of right now, only a small number of studies have demonstrated that ACT is beneficial in enhancing resilience^[59]^; however, the evidence is restricted because 2 of these studies were conducted in Persian,^[61–65]^ and none of this kind has been conducted in Nigeria except for the present study.

## 7. Future directions

Future research should address these limitations to provide more reliable insights into how effectively ACT supports employers and employees during difficult times. Improving ACT-based educational programs for restaurant and hotel managers is recommended. Implementing ACT programs in restaurant and hotel settings can lead to better service, reduced physical and mental exhaustion among professionals, and an overall improvement in service quality. These programs can be effective in boosting resilience and enhancing overall quality of life. Focusing on the positive relationships between accepting negative emotions, improving emotional tolerance, and reducing self-criticism can strengthen self-compassion and improve psychological well-being. Therefore, we suggest continuous application of psychological therapies such as acceptance and commitment therapy, cognitive behaviour therapy, and rational emotive behaviour therapy^[66–72]^ for restaurant and hotel workers is crucial, as they play a fundamental role in providing services and support to the community.

## Author contributions

**Conceptualization:** Joy Nneka Uchenye Ejikeme, John N. Igabari, Daphney Mawila-Chauke, Moses Onyemaechi Ede, Joy Oluchukwu Okoro, Blessing Ijeoma Attah, Eunice Ifenyinwa Ugwu, Joy Obiageli Oneli.

**Data curation:** Joy Nneka Uchenye Ejikeme, John N. Igabari, Moses Onyemaechi Ede, Blessing Ijeoma Attah, Eunice Ifenyinwa Ugwu.

**Formal analysis:** Joy Nneka Uchenye Ejikeme, John N. Igabari, Moses Onyemaechi Ede, Eunice Ifenyinwa Ugwu.

**Funding acquisition:** Joy Nneka Uchenye Ejikeme, John N. Igabari, Daphney Mawila-Chauke, Moses Onyemaechi Ede, Glory Mmachukwu Nwakpadolu, Joy Oluchukwu Okoro, Blessing Ijeoma Attah, Eunice Ifenyinwa Ugwu, Joy Obiageli Oneli.

**Investigation:** Joy Nneka Uchenye Ejikeme, John N. Igabari, Moses Onyemaechi Ede, Glory Mmachukwu Nwakpadolu, Joy Oluchukwu Okoro, Blessing Ijeoma Attah, Eunice Ifenyinwa Ugwu, Joy Obiageli Oneli.

**Methodology:** Joy Nneka Uchenye Ejikeme, John N. Igabari, Daphney Mawila-Chauke, Moses Onyemaechi Ede, Glory Mmachukwu Nwakpadolu, Joy Oluchukwu Okoro, Blessing Ijeoma Attah, Eunice Ifenyinwa Ugwu, Joy Obiageli Oneli.

**Project administration:** Joy Nneka Uchenye Ejikeme, John N. Igabari, Moses Onyemaechi Ede, Glory Mmachukwu Nwakpadolu, Joy Oluchukwu Okoro, Blessing Ijeoma Attah, Eunice Ifenyinwa Ugwu.

**Resources:** Joy Nneka Uchenye Ejikeme, John N. Igabari, Daphney Mawila-Chauke, Moses Onyemaechi Ede, Glory Mmachukwu Nwakpadolu, Blessing Ijeoma Attah, Eunice Ifenyinwa Ugwu.

**Software:** Joy Nneka Uchenye Ejikeme, John N. Igabari, Daphney Mawila-Chauke, Moses Onyemaechi Ede, Glory Mmachukwu Nwakpadolu, Blessing Ijeoma Attah, Eunice Ifenyinwa Ugwu.

**Supervision:** Joy Nneka Uchenye Ejikeme, John N. Igabari, Daphney Mawila-Chauke, Moses Onyemaechi Ede, Glory Mmachukwu Nwakpadolu, Joy Oluchukwu Okoro, Blessing Ijeoma Attah, Eunice Ifenyinwa Ugwu.

**Validation:** Joy Nneka Uchenye Ejikeme, John N. Igabari, Daphney Mawila-Chauke, Moses Onyemaechi Ede, Glory Mmachukwu Nwakpadolu, Blessing Ijeoma Attah, Eunice Ifenyinwa Ugwu, Joy Obiageli Oneli.

**Visualization:** Joy Nneka Uchenye Ejikeme, John N. Igabari, Daphney Mawila-Chauke, Moses Onyemaechi Ede, Glory Mmachukwu Nwakpadolu, Joy Oluchukwu Okoro, Eunice Ifenyinwa Ugwu, Joy Obiageli Oneli.

**Writing – original draft:** Joy Nneka Uchenye Ejikeme, John N. Igabari, Moses Onyemaechi Ede, Glory Mmachukwu Nwakpadolu, Joy Oluchukwu Okoro, Eunice Ifenyinwa Ugwu, Joy Obiageli Oneli.

**Writing – review & editing:** Joy Nneka Uchenye Ejikeme, John N. Igabari, Daphney Mawila-Chauke, Moses Onyemaechi Ede, Glory Mmachukwu Nwakpadolu, Eunice Ifenyinwa Ugwu, Joy Obiageli Oneli.
